# Unsupervised Domain Adaptation for Mitigating Sensor Variability and Interspecies Heterogeneity in Animal Activity Recognition

**DOI:** 10.3390/ani13203276

**Published:** 2023-10-20

**Authors:** Seong-Ho Ahn, Seeun Kim, Dong-Hwa Jeong

**Affiliations:** 1 Department of Artificial Intelligence, The Catholic University of Korea, Bucheon 14662, Republic of Korea; hohoan05@gmail.com; 2 School of Computer Science and Information Engineering, The Catholic University of Korea, Bucheon 14662, Republic of Korea; senniii9809@gmail.com

**Keywords:** animal activity recognition, wearable devices, unsupervised domain adaptation (UDA), deep adaptation network (DAN), domain adversarial neural network (DANN), deep reconstruction-classification network (DRCN)

## Abstract

**Simple Summary:**

This study aimed to improve animal activity recognition (AAR) using wearable sensor data, which often faces challenges due to sensor variability and individual variability across species. To address this problem, we adopted unsupervised domain adaptation (UDA) techniques to improve the AAR performance. The main objective of UDA is to enable AAR models to perform well on the newly enrolled unlabeled sensor datasets, even when they have different distributions than the pre-trained labeled datasets. The experiments performed with the dog and horse movement sensor datasets demonstrated that UDA significantly improved the classification performance by mitigating sensor variability and individual characteristics such as size, gender, and species. These findings highlight that UDA has practical applications in real-world scenarios where labeled data is scarce in certain measurement environments.

**Abstract:**

Animal activity recognition (AAR) using wearable sensor data has gained significant attention due to its applications in monitoring and understanding animal behavior. However, two major challenges hinder the development of robust AAR models: domain variability and the difficulty of obtaining labeled datasets. To address this issue, this study intensively investigates the impact of unsupervised domain adaptation (UDA) for AAR. We compared three distinct types of UDA techniques: minimizing divergence-based, adversarial-based, and reconstruction-based approaches. By leveraging UDA, AAR classifiers enable the model to learn domain-invariant features, allowing classifiers trained on the source domain to perform well on the target domain without labels. We evaluated the effectiveness of UDA techniques using dog movement sensor data and additional data from horses. The application of UDA across sensor positions (neck and back), sizes (middle-sized and large-sized), and gender (female and male) within the dog data, as well as across species (dog and horses), exhibits significant improvements in the classification performance and reduced the domain discrepancy. The results highlight the potential of UDA to mitigate the domain shift and enhance AAR in various settings and for different animal species, providing valuable insights for practical applications in real-world scenarios where labeled data is scarce.

## 1. Introduction

Behavior recognition of animals has emerged as a promising method for estimating their health status and monitoring abnormal behaviors. The widely-used types of sensors in identifying animal behavior include cameras [1,2,3] and wearable devices [4,5,6]. Wearable devices, comprising accelerometers, gyroscopes, magnetometers, pressure sensors, and global navigation satellite systems (GNSSs), have garnered increasing popularity in animal monitoring applications. Wearables offer numerous advantages, such as their lightweight nature, compact size, low power consumption, and ease of integration, making them highly suitable for recognizing animal behaviors [7,8]. In this regard, wearable sensors find applications in various contexts, including animal health and welfare [6,9], as well as smart animal farming [10,11] for monitoring animal behavior. For instance, an ML-based system was proposed to automatically classify behavioral patterns to estimate the health status or to detect animal illness [6,9]. The abnormal behavior of animals was identified as a means to enhance the economic efficiency of production and develop a smart farming system [10,11]. Moreover, wearable sensors can be effectively combined with other sensors, such as photoplethysmography, to measure cardiac diseases and other relevant health metrics [12].

Despite the advantages of wearable sensors, animal activity recognition (AAR) using inertial measurement units (IMUs), consisting of accelerometers and gyroscopes, encounters several obstacles such as different sensor positions, species variability, and annotation scarcity. One of the challenges in Animal Activity Recognition (AAR) is the variability introduced by different sensor positions. The back sensor is optimized for capturing larger movements, whereas the neck sensor is designed to detect intricate movements [13,14]. This variability is further exacerbated by differences in experimental settings and labeling criteria, which can diverge significantly among researchers [13,14,15]. In recent years, researchers have made efforts to tackle individual or sensor variability by leveraging state-of-the-art machine learning techniques [16,17]. One approach is transfer learning, which involves utilizing pre-trained knowledge constructed using one sensor for predicting AAR with another sensor. For instance, Kleanthous et al. (2022) address sensor heterogeneity and orientation placement in sheep activity recognition by utilizing convolutional neural networks (CNN) combined with hand-crafted features [16]. This approach enhances model generalization, especially concerning sensor orientation and position, and demonstrates the potential of transfer learning for the reusability of pre-trained models on the unseen data, achieving high accuracy rates across two different sensor types. While there have been some investigations into this aspect, comprehensive research remains limited.

The diversity in animal behavior, influenced by factors such as species, breeds, and sizes, presents a challenge in developing global models that can effectively generalize across different individuals or species [18,19,20,21,22]. A study by Ferdinandy et al. (2020) highlighted this challenge, reporting that cross-size validation, which categorizes dogs into small-sized (<10 kg), middle-sized (10–25 kg), and large-sized (>25 kg) groups, yielded a lower classification performance compared to the within-size validation settings [15]. Furthermore, the cross-species validation conducted by predicting wolves’ activities using an AAR model developed with the dog activity data underscores the variations. These variations can arise due to behavioral differences between breeds and species, as well as distinct body structures [15,18,19,20,22]. Despite the evident challenges, there remains a lack of studies addressing these issues in AAR.

The scarcity of annotation is an additional constraint in AAR. In most studies and public datasets, animal activities are annotated via the visual inspection of animal behaviors in video recordings, often requiring substantial human resources and potentially raising privacy concerns [5,17,21,22]. Federated learning, as proposed by Mao et al. (2022), provides a mechanism to update a global server model by aggregating only local parameters, eliminating the need to collect the raw data from local devices [17]. However, this approach still requires labeled data for each class to fine-tune the global model and customize individual models. Acquiring sufficient data for deep learning models is time-consuming, particularly given the unpredictable activities exhibited by animals, even those that are trained, such as dogs [13,14,15]. Consequently, the data collection and labeling process demands substantial domain knowledge, time, and resources, leading to limitations in the availability of large labeled datasets for supervised learning approaches [22]. To address these limitations and reduce the dependency on the labeled data, the adoption of unsupervised methods, such as domain adaptation methods, becomes more beneficial than traditional supervised approaches in enhancing AAR.

Unsupervised domain adaptation (UDA), a form of transfer learning, is a technique that reduces the variability between the labeled source data and the unlabeled target data in an unsupervised manner [23]. In human activity recognition (HAR), extensive research has been conducted to address the variability arising from diverse sensor placements by employing UDA techniques. For instance, Khan et al. (2018) introduced the HDCNN model, a transductive transfer learning approach tailored for convolutional neural networks, to facilitate HAR scalability without the need for extensive labeled data in the new domains [24]. To mitigate the challenges posed by sensor position variability in HAR, their approach demonstrated robustness in maintaining high accuracy across varying on-body sensor placements without necessitating extensive labeled data in the new domains. In addition, Sanabria et al. (2021) introduced ContrasGAN, an unsupervised domain adaptation technique for HAR that employs bi-directional generative adversarial networks and contrastive learning to address challenges in sensor data labeling [25]. The method was evaluated under various conditions, including cross-sensor transfer learning, demonstrating its effectiveness in adapting activity models across different sensor positions with a superior performance compared to the existing techniques. Meanwhile, Chang et al. (2020) conducted an in-depth exploration of unsupervised domain adaptation techniques in the context of sensor placement variability in HAR, highlighting the challenges in ensuring robustness against real-world heterogeneities in the sensor data [26]. While numerous studies in HAR have utilized unsupervised methods to circumvent the annotation challenge, AAR still faces this limitation, highlighting the imperative for further research in this area.

In this study, we employ unsupervised domain adaptation (UDA) techniques to address the challenges of domain variability in AAR without necessitating additional labeled data for new datasets. We employed three distinct types of UDA techniques, which have been widely adopted in the previous HAR studies. We utilized two public AAR datasets to explore the impact of UDA on domain variability between sensor positions, individual characteristics, and species. For the sensor position, we conducted domain adaptation between the neck and back sensors to reduce the sensor sensitivity depending on changes in the sensor position. We hypothesized that the activity patterns of dogs vary depending on the size (middle-sized and large-sized) and gender. Thus, to investigate the impact of these attributes, we categorized dogs into two groups, and these were used as the source and target domain, respectively. In the context of size, we segregated dogs into middle-sized and large-sized groups based on their weight. For gender exploration, we divided the dogs into male and female groups. Subsequently, we compared the classification results of three UDA techniques with those obtained when no domain adaptation technique was applied. In addition, we also evaluated inter-species variability by adapting the horse activity domain to the dog activity domain. In summary, our contribution can be described as follows:

1. UDA techniques demonstrate the potential to enhance the AAR performance by mitigating data heterogeneity arising from variations in individual behaviors and sensor locations.
2. UDA techniques exhibit versatility by being applicable to different mammal species, demonstrating their adaptability and applicability in the field of AAR.
3. We present empirical evidence that the influence of minimizing divergence-based, adversarial-based, and reconstruction-based UDA varies depending on the specific domain under consideration.

## 2. Materials and Methods

### 2.1. Datasets and Preprocessing

The public dataset of movement sensor data collected from 45 middle- or large-sized dogs was adopted for this study [13]. Their average age was 4.84 years (16–116 months) and the average weight was 24 kg (13–41 kg). The six-degree-of-freedom inertial measurement unit (IMU) sensors consisting of a 3-axis accelerometer and a 3-axis gyroscope (ActiGraph GT9X Link) were sampled at 100 Hz. The sensors were attached to the neck collar and the back belt of the harness to measure the dog’s behavior. The trained dogs were asked to perform three static tasks (sitting, standing, and lying down) and four dynamic tasks (trotting, walking, playing, and sniffing). They annotated each behavior on the movement sensor data by observing two videos recorded during the experiment. Only longer than one-second unambiguous and voluntary behaviors were annotated for extracting distinct canine behavioral patterns using movement sensors. In this study, we utilized six activity classes (lying on the chest, sitting, standing, walking, trotting, and sniffing) that contain a sufficient amount of data to build the AAR models. Consistent with the original study, each of the six IMU signals (3-axis accelerometer and 3-axis gyroscope signals) were segmented into two-second windows with a 50% overlap. Epochs shorter than 1 s and those with multi-label annotations were excluded from our analysis. The demographics of dogs and the amount of movement sensor data for each activity are described in Table 1.

Based on the experimental paradigm and the canine demographic of this dataset, we devised three scenarios to evaluate the UDA: (1) between the neck and back sensors, (2) between middle-sized and large-sized dogs, and (3) between male and female dogs. For the division of middle-sized and large-sized dogs by weight, we employed weight as the criterion, where dogs with a weight < 25 kg were categorized as middle-sized, and dogs with a weight ≥ 25 kg were considered large-sized [27,28].

To evaluate the cross-species adaptability of our proposed approach, we further utilized another animal movement database collected from horses [29]. This database contains 6-axis IMU data with a sampling rate of 100 Hz, similar to the dog database. In previous studies, although 17 different activities of 18 individual horses were labeled, only six classes from six individual horses were used due to data scarcity [17,30]. For the evaluation of UDA in this study, three classes (standing, walking, and trotting) from six horses were selected, as these activities commonly appeared in both the dog and horse databases. The total number of samples is 5374, 3353, and 27,210 for standing, walking, and trotting, respectively.

### 2.2. Proposed Framework

#### 2.2.1. Overview

**Figure 1:**
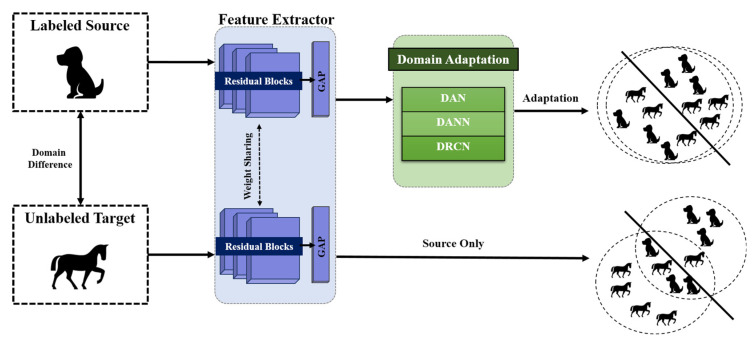
Overview of unsupervised domain adaptation framework (UDA). First, labeled source data was utilized to train residual network (ResNet) for extracting features for animal activity recognition. Then, unlabeled target data was used to predict animal activities using the model trained with source data (source only). To investigate the impact of UDA techniques that mitigate domain shift issues, the target data was evaluated after the implementation of three UDA techniques: deep adaptation network (DAN), domain adversarial neural network (DANN), and deep reconstruction-classification networks (DRCN).

To mitigate the heterogeneity between different domains in AAR, UDA adopts a cooperative training approach that leverages both the labeled data from the source domain and the unlabeled data from the target domain. For instance, if a dataset with labels is given, it plays a role as the source domain, while a newly acquired dataset without labels becomes the target domain. As depicted in Figure 1, UDA comprises residual networks (ResNet) and adversarial networks. The ResNet is widely recognized for its feature extraction capabilities, excelling not only in image classification but also in time-series data analysis. It is utilized for both the source and target domains, with their weights shared. Next, UDA techniques are employed to extract domain-invariant features from both the source and target domain. To examine the impact of UDA, the classification results of the target dataset after the implementation of UDA techniques were compared with those without UDA (source only). We employed three well-established UDA techniques that have been widely adopted in previous studies. These techniques include the deep adaptation network (DAN) for minimizing the divergence-based approach, the domain adversarial neural network (DANN) for an adversarial-based approach, and deep reconstruction-classification networks (DRCN) for a reconstruction-based approach.

#### 2.2.2. Unsupervised Domain Adaptation

Domain adaptation aims to improve the machine learning performance by alleviating the domain shift problem between the source and target domains, which exhibit significantly different data distributions [31]. This approach proves valuable in real-world applications where sufficient labeled data is available in one domain, but only unlabeled data exists in other domains. Given its nature as an unsupervised learning method, domain adaptation is widely applied in computer vision [32,33] and natural language processing [34].

In activity recognition using wearable devices, domain specificity is highly influenced due to sensor variability [26,35]. Furthermore, AAR faces significant challenges related to domain shift problems, primarily caused by individual variability arising from factors such as size, species, and measurement environments [8,16,19]. In addition, labeling animal activities with real-time sensor data poses considerable difficulty. Thus, unsupervised domain adaptation methods, where the label of the target domain is not required for learning, offer a promising solution to address these limitations and enable effective AAR in diverse settings.

Domain-Invariant Feature Learning (DIFL) is a widely employed domain adaptation methodology that has been applied across various fields. The primary objective of DIFL is to extract features that are both predictive and invariant to domain differences, utilizing methodologies such as minimizing divergence, adversarial learning, and reconstruction [23]. In this study, to identify the most suitable domain adaptation approach for AAR, we compared three types of DIFL methodologies: DAN for minimizing the divergence-based approach, DANN for an adversarial-based approach, and DRCN for a reconstruction-based approach.

**Figure 2:**
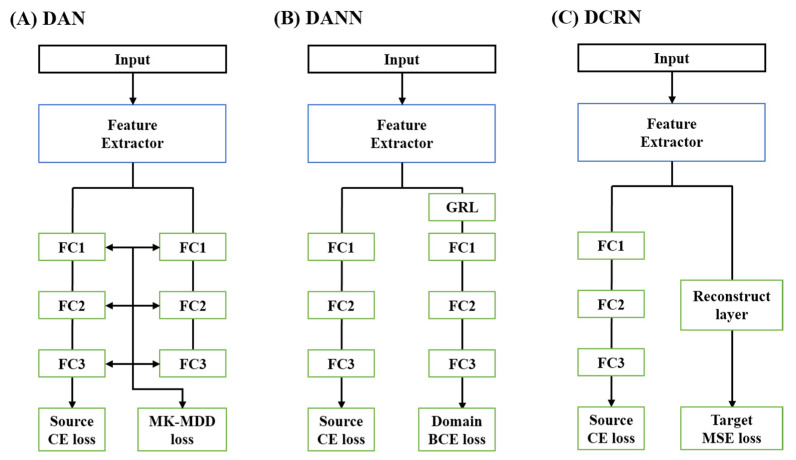
Model architecture of three different types of unsupervised domain adaptation. (**A**) Deep adaptation network (DAN) is a minimizing divergence-based approach, (**B**) domain adversarial neural network (DANN) is an adversarial-based approach, and (**C**) deep reconstruction-classification networks (DRCN) are a reconstruction-based approach. FC: fully-connected layer, CE: cross entropy, MK-MDD: multiple kernel variant of maximum mean discrepancy, GRL: gradient reversal layer, BCE: binary cross entropy.

Methods based on minimizing divergence aim to reduce the variance between the source and target domains. The DAN [36] employs the multiple kernel variant of the maximum mean discrepancy (MK-MMD) to compute the variance between the two domains (Figure 2A). By minimizing this variance while ensuring accurate class predictions in the source domain, DAN explicitly reduces the domain differences, enhancing the transferability of features in task-specific layers. The MK-MMD, in this process, explicitly aligns the hidden state representations from different domains [36]. The loss function for DAN is given by:

(1) $${Loss}_{DAN}=CE\left(f\left(x_{s}\right),y_{s}\right)+\lambda \sum_{n}D(f^{n}(x_{s}),\;f^{n}(x_{t})),$$

where $x_{s}$ and $x_{t}$ are the inputs from the source and target domains, $y_{s}$ is the label of source domains, $f^{n}$ are the hidden states of *n*th layer for the classifiers, CE is the cross-entropy, respectively. The function D represents MK-MMD and the parameter λ is the trade-off parameter.

Next, the DANN [37] is an adversarial-based domain adaptation method renowned for its superior performance across diverse domains and tasks, especially those involving the time series data [38,39]. DANN employs a gradient reversal layer (GRL) to discern whether the current data belongs to the source or target domain (Figure 2B). The feature extractor is trained to ensure that this distinction is indistinguishable, thereby facilitating the learning of domain-invariant features. The GRL negates the gradient of the domain classifier during backpropagation, making it challenging to determine whether the training data belongs to the source or target. The loss function for DANN is given by:

(2) $${Loss}_{DANN}=CE\left(f\left(x_{s}\right),y_{s}\right)+\lambda (BCE\left(g\left(x_{s}\right),\;d_{s}\right)+BCE\left(g\left(x_{t}\right),d_{t}\right)),$$

where functions *f* and *g* denote the class classifier and the domain classifier, while $d_{s}$ and $d_{t}$ are the domain labels for the source and target domains, and BCE is the binary cross-entropy respectively.

Last, reconstruction-based methods aim to achieve domain adaptation by learning representations that can accurately classify the source domain and effectively reconstruct the source or target domain. The DRCN [40] accomplishes this by classifying the source domain while reconstructing the target domain (Figure 2C). The loss function for DRCN is given by:

(3) $${Loss}_{DRCN}=CE\left(f\left(x_{s}\right),y_{s}\right)+\lambda (MSE\left(h\left(x_{t}\right),x_{t}\right)),$$

where *h* is the decoder for reconstructing the target domain data and MSE denotes a mean squared error. 

For a comparative performance evaluation of the three DA techniques, we standardized the hyperparameters. The hidden states of the fully connected layers for DAN, DANN, and DRCN were consistently set to 128, 64, and the number of classes, respectively. The reconstruction pipeline of DRCN was symmetrically constructed using transposed convolution layers, which is consistent with the original paper [40].

#### 2.2.3. Residual Neural Network (ResNet)

The results of a previous study [37] have shown that the classification performance of domain adaptation highly depends on the performance of the feature extractor. We propose a feature extractor based on ResNet [41,42] to achieve a good performance on the time series data. ResNet was originally designed as an architecture for the image classification task, and it has achieved a high performance in image classification by preventing the gradient vanishing that occurs when layers are deeply stacked via residual connections. However, previous research [42,43] has validated that ResNet-based architecture outperforms equally well on time series classification tasks, including wearable sensor-based activity recognition applications. [44,45,46]. The HAR studies that adopt UDA utilized CNNs as feature extractors because CNNs are computationally more efficient than the recurrent neural networks [24,26]. This ensures the model’s capability not only within our experimental setting but also in other real-world applications.

In the feature extractor, we implemented a residual block which set up the kernel size {(1, 7), (1, 5), (1, 3)}, and applied batch normalization and Leaky ReLU as the activation functions. We stacked three residual blocks with the number of kernels {64, 128, 128}. The output of the last residual block is applied to the global average pooling to reduce the dimension of the hidden state. In the label classifier, we used two fully connected layers on the hidden state {128, 64} with batch normalization, leaky ReLU, and a dropout layer. The hidden state of the final fully connected layer is the number of classes.

### 2.3. Experimental Setting

All experiments were conducted in the local environment, employing a 12th Gen Intel^®^ Core™ i5-12400F processor and an NVIDIA GeForce RTX 3070 Ti. To implement our proposed model, PyTorch was utilized with Python 3.9, and for reproducibility, random states were fixed. We employed the Adam optimizer with a learning rate of 0.00001 and a weight decay of 0.0001 while setting the batch size to 256, dropout to 0.2, and λ to 1.0. 

For training and evaluating each UDA model, the 2 s segments were partitioned into training and test sets with an 8:2 ratio for each UDA model. Additionally, the training set was further divided into training and validation sets with a ratio of 3:1. To assess the entire dataset, the five-fold cross-validation procedure was performed by repeating this procedure five times. In the within-setting and without-UDA setting, we selected the best model based on its accuracy in the source validation set and evaluated its performance on the target test set. In the domain adaptation setting, we reported the best performance achieved on the target test set during 100 epochs.

To compare the classification performance of each UDA method, AAR was evaluated using accuracy, which measures the overall performance, and the F1 score, which considers both precision and recall, as defined with the following equations:

(4) $$Accuracy=\frac{TP+TN}{TP+FP+TN+FN},$$

(5) $$Precision=\frac{TP}{TP+FP},$$

(6) $$Recall=\frac{TP}{TP+FN},$$

(7) $$F1-score=\frac{2\times Recall\times Precision}{Recall+Precision},$$

where *TP*, *TN*, *FP*, and *FN* are the number of true positives, true negatives, false positives, and false negatives, respectively.

## 3. Results

### 3.1. Classification Results

Using the dog movement dataset [13], we evaluated to test the hypothesis that UDA can alleviate the domain shift problem caused by the sensor position (back (B) vs. neck (N)) and canine characteristics (middle-sized (MS) vs. large-size (LS) and male (M) vs. female (F)). Table 2 presents the classification performance of the unlabeled data in the target domain using the trained source model with and without the domain adaptation technique. The within-domain performance by predicting the source data are also presented in Table A1 in Appendix A. The results show that applying domain adaptation to mitigate the sensor variability improved the classification performance over not using UDA for all three DIFL methods. Specifically, DAN exhibited the most significant enhancement in the classification performance. When using back sensors as the source domain and neck sensors as the target domain (B→N), both classification accuracy and the F1 score showed enhancements of 16.62% (60.88%→77.50%) and 17.10% (45.85%→62.95%), respectively. Similarly, in the N→B scenario, DAN improved the accuracy by 14.41% (65.74%→80.15%) and the F1 score by 23.11% (46.44%→69.55%).

**Figure 3:**
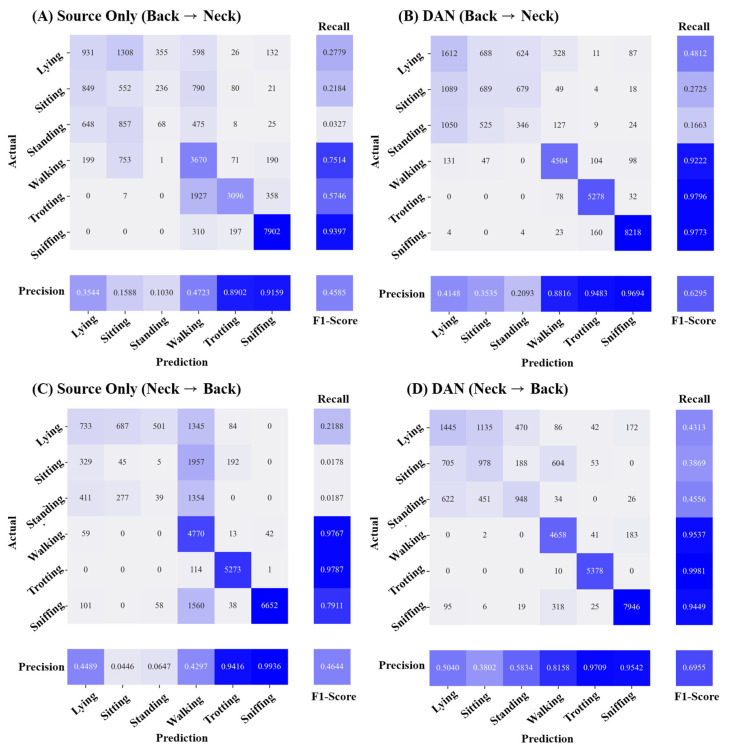
Confusion matrix of AAR classification depending on domain adaptation between neck and back sensors. The left insets (**A**,**B**) represent classification results without applying domain adaptation (source-only) in the neck→back scenario and the back→neck scenario, respectively. The right insets (**C**,**D**) represent classification results after applying deep adaptation network (DAN) in the neck→back scenario and the back→neck scenario, respectively. The deeper blue signifies a greater number of true positives, indicating higher classification performance for each class.

Figure 3 depicts the confusion matrix illustrating the classification results for six AAR classes. The left insets (Figure 3A,C) show the classification results for the source only case, while the right insets (Figure 3B,D) show the results after applying the domain adaptation with DAN, which exhibited the highest classification performance. Across both sensors, DAN enhanced the recall and precision for all six AAR classes, leading to significant increases in the F1 score. The activity that was consistently well classified was sniffing, regardless of the application of domain adaptation. On the other hand, static movements, including lying on the chest, sitting, and standing, exhibited a poor classification performance due to their similarity. Frequent misclassification between walking and trotting were observed in the B→N scenario. The application of DAN mitigated the misclassification issue occurring from the similarity between these activities. In the B→N scenario, recall and precision for predicting the walking activity increased by 17.08% and 40.93%, respectively. While the classification performance of the three static movements also improved after applying DANN, the problem of misclassification among the static activities remained a challenge.

**Figure 4:**
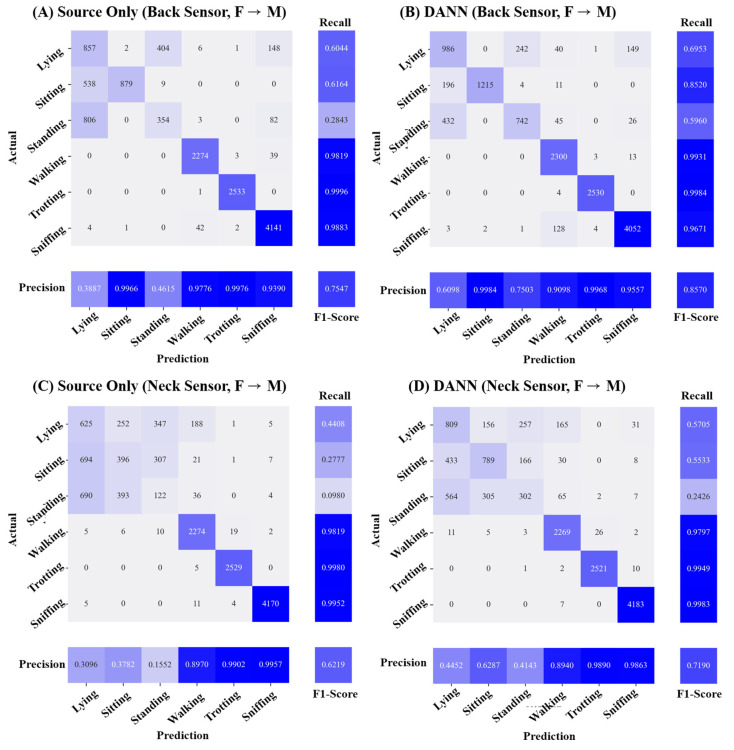
Confusion matrix of AAR classification depending on domain adaptation for gender. The left insets (**A**,**B**) represent classification results in the female→male scenario with back sensors, where domain adaptation was not applied (source-only). The right insets (**C**,**D**) represent classification results in the female→male scenario with neck sensors, where domain adversarial neural network (DANN) was employed. The deeper blue signifies a greater number of true positives, indicating higher classification performance for each class.

Next, we experimented to verify the feasibility of domain adaptation between the sizes and genders. The overall classification performance using the back sensor was higher than when using the neck sensor, regardless of applying the domain adaptation. We found that the application of domain adaptation led to improvements in the classification performance in all cases. A greater improvement was observed when the domain adaptation was performed concerning gender compared to size. In particular, when using the back sensor, applying DANN for domain adaptation between males and females resulted in an approximate 7.65% increase (81.22%→88.87%) in the F1 score for the M→F scenario and a 10.23% increase (75.47%→85.70%) in the F1 score for the F→M scenario. Similarly, when using the neck sensor, DANN demonstrated the most improved classification performance, with a 9.71% increase (62.19%→71.90%) in the F1 score in the F→M scenario. In the M→F scenario, however, DAN achieved the highest classification performance, but the F1 score only exhibited slight improvements, surpassing source-only and DANN by 2.04% and 1.85%, respectively. Figure 4 illustrates that the classification performance for each activity followed a similar trend as that of the sensor adaptation classifiers, with large misclassifications between static activities.

Meanwhile, for both the back and neck sensors, there was no consistent result with regard to the domain adaptation techniques. In the MS→LS scenario, DRCN exhibited the best classification performance, resulting in a 5.75% increase and a 3.06% increase in the F1 score when utilizing the back and neck sensors, respectively. However, in the LS→MS scenario, most UDA techniques failed to achieve significant improvements in the classification performance. Specifically, when utilizing the neck sensors, the highest accuracy was observed when domain adaptation was not applied (source only).

**Figure 5:**
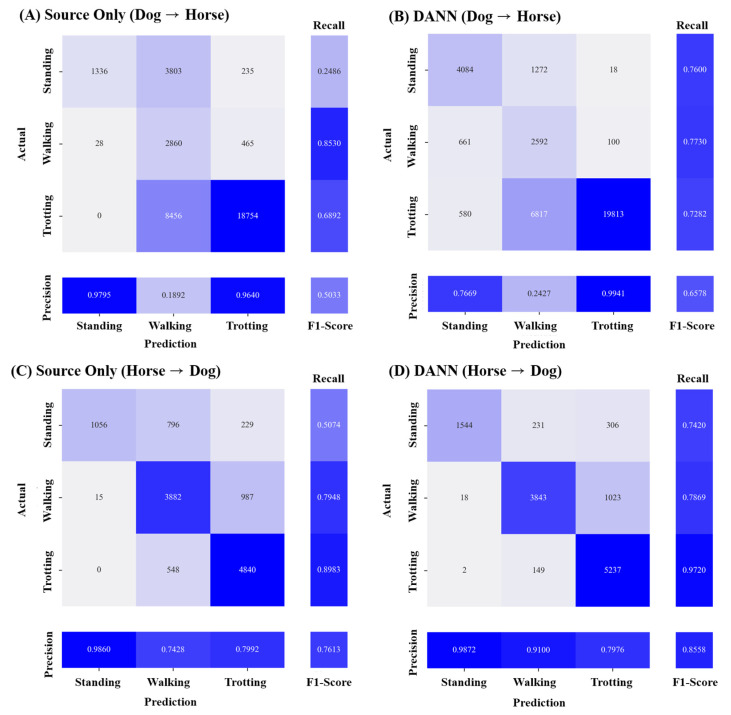
Confusion matrix of AAR classification depending on domain adaptation for species. The left insets (**A**,**B**) represent classification results in the dog→horse scenario and the horse→dog scenario, respectively, without the application of domain adaptation (source-only). The right insets (**C**,**D**) represent classification results in the dog→horse scenario and the horse→dog scenario, respectively, after the application of domain adversarial neural network (DANN). The deeper blue signifies a greater number of true positives, indicating higher classification performance for each class.

To investigate the potential of domain adaptation in mitigating domain shifts between species, we incorporated the movement sensor data collected from horses in addition to the dog dataset. Since the animal activity classes differed between the horse and dog datasets, we focused on the three common classes: standing, walking, and trotting. Additionally, as horse activities were only measured on the neck, we performed the comparison using the neck sensor data. In both scenarios involving the use of horse data as the target domain (D→H) and the source domain (H→D), DANN consistently resulted in the most substantial improvements in the classification performance. In the D→H scenario, the application of DANN resulted in an F1 score increase of 15.45% (50.33%→65.78%). Similarly, when employing DANN for H→D, it led to an enhanced performance, showing an F1 score increase of 9.45% (76.13%→85.58%). Notably, despite the application of domain adaptation techniques, the classification performance for the walking activity was lower in the D→H scenario compared to that in the H→D scenario (Figure 5). These results demonstrate that when predicting the dog movement data using the classification model’s trained horse movement data (H→D), the classification performance was higher compared to the D→H scenario.

### 3.2. Latent Space Analysis

The influence of domain adaptation on the classification performance was further examined qualitatively using t-distributed stochastic neighbor embedding (t-SNE), a widely adopted nonlinear dimensionality reduction technique for visualizing high-dimensional data in a low-dimensional space [47]. It constructs a joint probability distribution of high-dimensional vectors, assigning higher probabilities to similar vectors and lower probabilities to dissimilar vectors. Subsequently, it establishes a similar probability distribution in the low-dimensional map by minimizing the Kullback–Leibler divergence between the joint probability distributions of the low-dimensional and high-dimensional space. To reduce the high-dimensional latent vectors, which were used for predicting the activity labels, into a two-dimensional space, the principal component analysis (PCA) was applied to the latent vectors just before the softmax layer.

**Figure 6:**
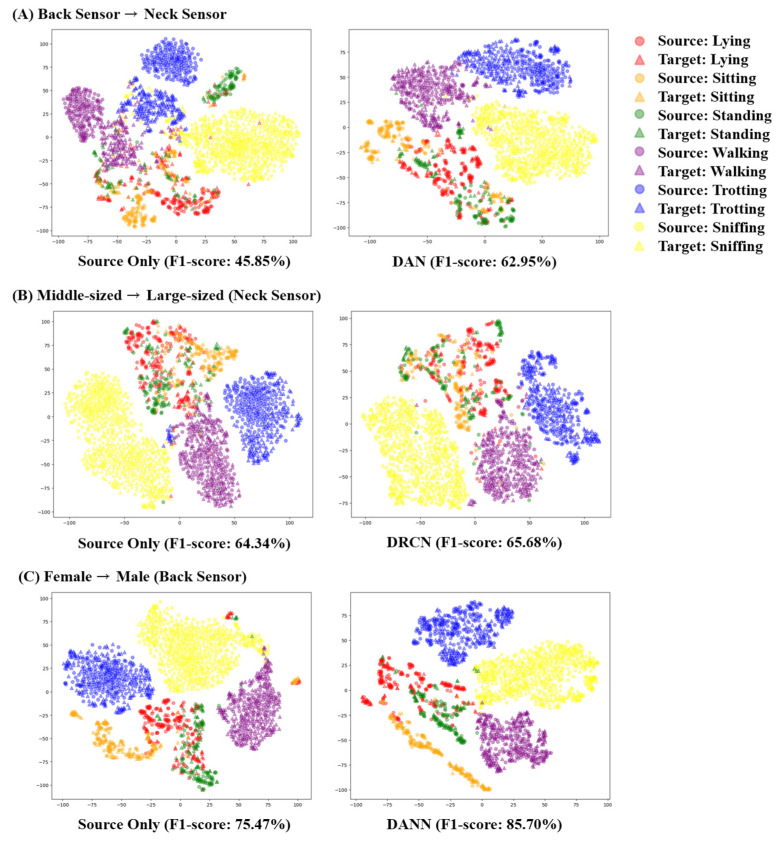
Visualization of t-SNE results of dog activity classification. The (**left**) columns show results without employing domain adaptation, whereas the (**right**) columns show results from the most successful among three domain adaptation techniques. The circles represent features from the source domain and the triangles are features from the target domain.

Figure 6 demonstrates the t-SNE visualization of feature vectors from both the source domain (represented as circles) and the target domain (represented as triangles). In the scenario where the back sensors were used to train the AAR classifier and the neck sensors were used for prediction (B→N), the features of the back and neck sensors were noticeably separated for both the walking (represented with purple color) and trotting class (represented with blue color), as shown in the left inset of Figure 6A. On the other hand, the application of DAN aligned the domain-shifted features of these classes, resulting in a 16.87% improvement in the F1 score, as shown in the right inset of Figure 6B. Additionally, a considerable number of neck sensor features in the standing class (represented as green triangle) were separated from the cluster of back sensor features (represented as green circle) without employing any domain adaptation techniques. The domain-shift problems between the source and target domain were mitigated after the implementation of DAN, leading to an improved classification performance. Nevertheless, there remains a considerable number of misclassifications between the standing and other static classes, namely, lying on the chest and sitting.

Domain adaptation with respect to the size did not result in a significant improvement of the classification performance since non-static activities including walking, trotting, and sniffing were well-classified even before the implementation of domain adaptation techniques. As shown in Figure 6B, the DRCN, which increased the F1 score by 1.34%, did not resolve the misclassification issue between static activities. This is primarily due to the low classification performance between static movements of the source model. In the scenario of adapting the female domain to the male domain, DANN, which led to a 9.73% improvement in the F1 score, alleviated the domain-shift issue within the static activity classes. In Figure 6C, the t-SNE visualization demonstrates this issue, where certain portions of lying on the chest (red triangle), standing (green triangle), and sitting (orange triangle) classes in the target domain were initially mapped closely to the sniffing (yellow circle) or walking activity (purple circle) classes in the source domain prior to the application of domain adaptation. Applying DANN provides a better feature representation of these classes by aligning the target domain data with the source domain data, resulting in an enhanced classification performance.

**Figure 7:**
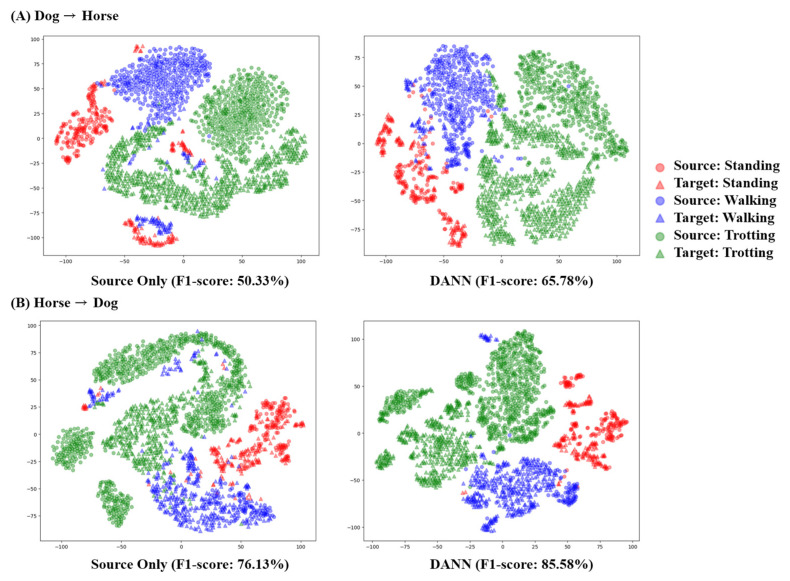
Visualization of t-SNE results for interspecies activity classification. The (**left**) columns show results without employing domain adaptation, whereas the (**right**) columns show results from domain adversarial neural network (DANN), the best performing among three domain adaptation techniques. The circles represent features from the source domain and the triangles are features from the target domain.

In the scenario of AAR classification across species, domain-shift problems became more conspicuous. In the left inset of Figure 7A, before the implementation of domain adaption techniques, it was observed that features in the source domain, depicted as circles, were positioned at the upper portion, while those in the target domain, depicted as triangles, were positioned at the lower portion. Additionally, the standing and walking samples in the target domain exhibited a noticeable segregation in both Figure 7A,B. These observations could be associated with a poor classification performance, as a majority of samples from the standing and trotting classes were misclassified as a walking class. As shown in the right insets of Figure 7, the application of DANN mitigates the segregation within the same classes by aligning the target domain with the source domain, increasing the F1 score by 13.87% and 9.27% for the dog→horse and horse→dog scenarios, respectively.

## 4. Discussion

This study aims to explore the influence of addressing domain variability, commonly encountered in AAR, by leveraging three types of UDA techniques, without the need for additional labeled data in novel datasets. As a result, the domain adaptation techniques demonstrated its potential to enhance the AAR performance by mitigating data heterogeneity arising from variations in individual behaviors and sensor locations. When dealing with newly enrolled individuals or species for efficient AAR, the importance of global models becomes evident. For instance, canine behaviors may differ based on factors such as weight, gender, and age [18,19,20]. UDA techniques prove to be particularly valuable in real-world applications where manual labeling is challenging, as it does not require any labels in novel datasets, simplifying the training of personalized classifiers. The findings of this study demonstrate the versatility and adaptability of UDA in the field of AAR, as it can be applied to diverse individuals and species.

As hypothesized, variations in canine characteristics can influence activity classification, attributed to the differences in sensor locations, velocities, behavioral patterns, and anatomical structures [13,15,18,19,20]. We found that UDA effectively performed across all domains, encompassing sensor locations, gender, and size (Table 2). We examined the impact of canine characteristics on the success of domain adaptation by assessing the domain adaptation performance that appears differently.

UDA had a significant impact on reducing sensor variability, particularly when employing DAN, which is a minimizing divergence-based UDA technique. In the field of human activity recognition, domain adaptation techniques have been employed to apply pre-trained classifiers from one wearable device (e.g., smartphone) to predict activities using other wearable devices (e.g., smartwatches) [24,25,26]. Specifically, in a comparative study examining the influence of various UDA approaches on sensor heterogeneity in HAR, Chang et al. (2020) reported that feature matching, a minimizing divergence-based approach, exhibited a more significant enhancement of the classification performance compared to confusion maximization, an adversarial-based approach [26]. Similarly, we found the most significant improvement in the classification performance within the DAN results compared to DANN and DRCN in the context of domain adaptation between the back and neck sensors.

Our results imply that UDA can be leveraged in AAR when novel wearable devices are introduced in different environments. This versatility is crucial in real-world scenarios, as AAR has garnered increased attention due to concerns for animal welfare and the need to enhance livestock productivity, while lots of commercial wearable devices have been developed. Moreover, these sensors can be attached to various body parts depending on the purpose of the measurement, environmental conditions, and animal characteristics. Neck sensors are widely used in AAR due to their ease of attachment to animals [4,5,6,7,8,9,10,13,14,15,16,17]. However, neck sensors have shown a lower classification performance due to orientation shifts caused by the tightness of sensors [13,14,48]. Our findings in Figure 3 and Figure 4 are consistent with previous studies, which also reported lower classification results when predicting animal activities with the neck sensor data. One of the primary reasons for the decreased performance with the neck sensors in our study lies in the misclassification between static behaviors, namely standing, sitting, and lying on the chest. These behaviors share high interactivity similarity as they provide limited information, which is primarily related to sensor position, and possibly minor details regarding velocity and acceleration. The individual variability in the sensor position, which is attributed to size and anatomical structure differences, could exacerbate these errors. On the other hand, the back sensor proves to be more suitable for capturing these static behaviors because of its stability, but the neck sensor remains crucial since it is commonly used in practical AAR applications. For example, for capturing various activities of animals, the movement of the neck becomes more important, particularly concerning behaviors such as eating and drinking behavior [13].

In the domain adaptation concerning gender and size, we observed a more pronounced influence of domain adaptation in cases involving gender compared to those involving size. Previous studies have reported behavioral differences between male and female dogs linked to factors such as aggressiveness, sociability, and anatomical structure [18]. However, there was no study on AAR related to this aspect of our knowledge. Given that there exists numerous studies utilizing the IMU sensor data for gender classification in human activity recognition [49,50], our finding presents a novel perspective by examining the differences in activity patterns between male and female dogs. In contrast to the results observed in sensor adaptation, DANN resulted in the most significant improvement when compared to DAN and DRCN.

On the other hand, the canine size was not notably affected by the use of UDA. In general, dog breeds can be categorized into small-sized, medium-sized, and large-sized based on their weights, although there are controversies regarding the global standard. This categorization of dogs remains relevant due to its impact on behavior recognition. For example, some studies have indicated that large-sized dogs exhibit distinct behavior patterns attributed to factors such as aggressiveness and sociability [18,19]. Although DRCN demonstrated an improved classification performance in the middle-sized→large-sized scenario, the enhancement was not significant compared to the source-only cases and other UDA techniques. Furthermore, in the large-sized→middle-sized scenario, the classification performance of the source-only case, without any application of UDA techniques, surpassed that of the use of UDA techniques. This result can be attributed to the database being collected from only middle-sized and large-sized dogs, aiming to achieve a more homologous dataset with reduced variability. However, the use of domain adaptation would be more valuable when collecting movement datasets of small-sized dogs, as it would help bridge the gap caused by the differences in size and the behavioral patterns between small-sized and large-sized dogs. Ferdinandy et al. (2020) reported that individual variability depending on the size (small-sized, medium-sized, and large-sized) significantly influences the AAR performance [15]. Considering their work on cross-species using the movement sensor data of wolves, it would be appropriate to apply and evaluate our approach. Unfortunately, while they publicly provided the dataset, we could not train UDA models using this data due to the data scarcity and class imbalance. Another publicly available dataset was provided by Marcato et al. (2023), where they evaluated the inter-breed AAR model by collecting data from Labrador Retrievers, Golden Retrievers, and their crosses [14]. However, the performance of intra-breed and inter-breed classification did not show significant differences since these breeds had similar sizes and traits. Furthermore, we could not apply UDA techniques concerning age, despite its potential high influence on behavioral patterns, as the database only provides data from adult dogs. For example, puppy and juvenile canine behavior can differ according to their development stages, while senior canine behavior can differ due to aging [51,52].

The domain adaptation between the horse activity dataset and the dog activity dataset demonstrated that UDA can alleviate the domain-shift issue in inter-species AAR. Specifically, DANN exhibited the most significant enhancement in the classification performance, increasing the F1 score by 15.45% in the dog→horse scenario and 9.45% in the horse→dog scenario, respectively. Interestingly, similar to the results in gender adaptation, DANN, an adversarial-based UDA, outperformed DAN, a minimizing divergence-based one that showed the most substantial impact on sensor adaptation. In the HAR study conducted by Chang et al. (2020), adversarial-based UDA outperformed other UDA techniques in specific experimental settings, such as UDA between the foot and wrist wearable sensors, while a minimizing divergence-based UDA exhibited the most pronounced impact in the majority of sensor adaptation scenarios [26]. The authors suggest a plausible interpretation that adversarial-based UDA techniques are effective when dealing with big domain shift issues. Similarly, in our study, which involves big domain shifts between horse and dog datasets, encompassing interspecies differences and varying experimental settings, we found that DANN was more effective in alleviating these shifts compared to DAN.

Although the domain shift issues involving sensor positions, canine characteristics, and interspecies variability were mitigated with the adoption of domain adaptation techniques, there are some challenges in the application of UDA in AAR: the low classification performance of static movements. As shown in Figure 6, the static movements including lying on the chest, sitting, and standing were indistinguishable with t-SNE visualization. Although the implementation of UDA techniques lessened this issue by mapping the target domain to the source domain, it could not resolve the misclassification between static movements.

It is important to note that the success of the domain adaptation technique heavily relies on the performance of the feature extractor in the source domain. Since the main idea behind this technique involves adversarial learning to confuse the models between the source and target domain in an unsupervised manner, it does not inherently extract novel information from the novel datasets. This limitation has been observed in previous studies that exhibited considerable performance differences between using AlexNet and ResNet as feature extractors in CDAN [37]. This highlights the significance of utilizing a feature extractor capable of effectively distinguishing static movements. While conventional knowledge-based feature extraction methods, including temporal and statistical features, have provided good results, they often depend on researcher-defined features, making them less suitable for generalized models. In the context of AAR, where diverse domains are encountered, automatic feature extraction is more suitable for adapting to novel and unsupervised datasets. Therefore, we opted for an end-to-end model for our framework because it allows for adaptive feature extraction from novel measurement settings, facilitating the ease of implementation and flexibility. For this study, we employed ResNet, a type of CNN that is well-suited for time series data and is commonly used as a standard model, as our feature extractor [42]. However, it is essential to further improve the feature extractor in UDA to construct a robust model. Another possible solution for improving the classification performance of static movements is to build multimodal models combined with different sensors. For instance, computer vision techniques can be fusioned because the postures of animals can be more distinguishable with camera-based modality compared to wearable devices [22,53].

Data scarcity remains a significant factor limiting the use of deep learning methods in AAR. In the domain adaptation regarding canine size and species, a lower classification performance was observed when the number of samples in the source domain was limited. Specifically, the large-sized dog group, which exhibited no influence of any UDA techniques, had a substantially smaller number of samples for the static movements (lying on the chest, sitting, and standing) compared to those in the middle-sized dog group (Table 1). Similarly, when dog activities is considered the source model, which contained fewer samples compared to horse activities, a notable amount of misclassification persisted within the walking class, even with the implementation of DANN (Figure 5). Therefore, it is necessary to acquire large datasets encompassing broader ranges of activities, populations, and species with a sufficient number of samples to enable the development of a more generalized model.

With the advancement of digital technology and the growth of the pet market, wearable devices have emerged to enhance productivity and animal welfare. Therefore, it is anticipated that more public datasets will become available, encompassing various species and sensors in diverse environments. In this context, UDA would prove more beneficial in constructing a generalized AAR model and facilitating a better understanding of behavioral patterns to enhance animal welfare and productivity. In future studies, it is essential to conduct empirical studies that assess the practical applicability of UDA in online prediction for diverse livestock using a source model constructed from labeled offline datasets.

## 5. Conclusions

This study explored the effectiveness of unsupervised domain adaptation in mitigating the domain shift problem in animal activity recognition using wearable sensor data. Through extensive experiments with movement sensor datasets of dogs and horses, we demonstrated that UDA aligns domain-invariant features of the target domain with those of the source domain, leading to an improved classification performance. The results showed that applying UDA not only enhanced the classification performance within the same species but also across different species. Notably, DAN exhibited the most pronounced effect in mitigating sensor variability, whereas DANN had the most significant impact in interspecies domain adaptation. In conclusion, UDA proves to be effective in effectively addressing sensor variability and individual characteristics, making it a promising solution for the real-world application of AAR in diverse settings with the limited availability of labeled data.

## Figures and Tables

**Table 1:** Description of canine movement dataset used for unsupervised domain adaptation.

		Size		Gender		Total
		Middle-Sized	Large-Sized	Male	Female	
Demographics of Dogs	Number of Dogs (Male)	23 (10)	22 (12)	22	23	45 (22)
	Age (Months)	61.3	54.6	55.64	60.35	58
	Weight (kg)	18.7	30.0	25	23.48	24.0
Amount of Samples	Lying on chest	2327	1023	1418	1932	3350
	Sitting	2273	255	1426	1102	2528
	Standing	1443	638	1245	836	2081
	Walking	2953	1931	2316	2568	4884
	Trotting	3651	1737	2534	2854	5388
	Sniffing	4926	3483	4190	4219	8409
	Total	17,563	9067	13,129	13,511	26,640

**Table 2:** Comparison of classification performance depending on the presence of domain adaptation. The numbers in bold represent the highest classification performance achieved among classifier without any domain adaptation technique and three classifiers employing various UDA methods. DAN: Deep Adaptation Network, DANN: Domain-Adversarial Training of Neural Networks, DRCN: Deep Reconstruction-Classification Networks, S: Source, T: Target, B: Back sensor, N: Neck sensor, MS: Middle-sized, LS: Large-sized, M: Male, F: Female, D: Dog, H: Horse.

Adapted Domain	Sensor Positions	S→T	Source Only		DAN		DANN		DRCN	
			Accuracy	F1 Score	Accuracy	F1 Score	Accuracy	F1 Score	Accuracy	F1 Score
Sensors		B→N	0.6088	0.4585	**0.7750**	**0.6295**	0.6961	0.5420	0.6947	0.5269
		N→B	0.6574	0.4644	**0.8015**	**0.6955**	0.7640	0.6353	0.7616	0.6562
Size	Back	MS→LS	0.8771	0.7251	0.8532	0.7364	0.8788	0.7559	**0.8890**	**0.7826**
		LS→MS	0.8392	0.7874	0.8032	0.7267	**0.8433**	**0.7891**	0.8387	0.7800
	Neck	MS→LS	0.8496	0.6435	0.8352	0.6487	0.8476	0.6476	**0.8592**	**0.6741**
		LS→MS	**0.7655**	**0.6465**	0.7621	0.6291	0.7627	0.6244	0.7531	0.6027
Gender	Back	M→F	0.8866	0.8122	0.9269	0.8805	**0.9297**	**0.8887**	0.9274	0.8845
		F→M	0.8407	0.7547	0.8832	0.8271	**0.9007**	**0.8570**	0.8881	0.8376
	Neck	M→F	0.7968	0.6419	**0.8073**	**0.6623**	0.8015	0.6438	0.8015	0.6505
		F→M	0.7705	0.6219	0.8174	0.7012	**0.8282**	**0.7190**	0.8104	0.6828
Species	Neck	D→H	0.6386	0.5033	0.7339	0.6234	**0.7371**	**0.6578**	0.6854	0.6467
		H→D	0.7915	0.7613	0.7797	0.7951	**0.8600**	**0.8558**	0.8338	0.8260

## Data Availability

No new data were created in this study.

## Appendix A

Since the data in the source domain were also partitioned into a training set and test set with a ratio of 8:2, the model trained on the labeled data of the source domain is evaluated with the test data in the source domain which were excluded during the training process. Thus, we name this evaluation a within-domain classification.

**Table A1:** Classification performance of within-domain classification by evaluating the source classifier with the source data.

Domain	Sensor Positions	Source	Within-Domain Classification			
			Accuracy	Precision	Recall	F1 Score
Sensors		Back	0.9794	0.9670	0.9663	0.9666
		Neck	0.9272	0.8767	0.8709	0.8734
Size	Back	Middle-sized	0.9778	0.9653	0.9671	0.9661
		Large-sized	0.9785	0.9613	0.9573	0.9592
	Neck	Middle-sized	0.9346	0.9031	0.9058	0.9037
		Large-sized	0.9537	0.8644	0.8304	0.8374
Gender	Back	Male	0.9787	0.9678	0.9688	0.9677
		Female	0.9834	0.9731	0.9702	0.9716
	Neck	Male	0.9459	0.9165	0.9125	0.9140
		Female	0.9452	0.8979	0.8967	0.8972
Species	Neck	Dog	0.9951	0.9960	0.9918	0.9939
		Horse	0.9982	0.9950	0.9950	0.9950
